# A Text Book of Human Physiology

**Published:** 1876-02

**Authors:** 


*A Text-Book of Human Physiology, designed for the use of Practitioners and Students of Medicine.* By AUSTIN FLINT, Jr., M. D. New York: D. Appleton & Co., 1876. Buffalo: Martin Taylor.

In this volume Dr. Flint has condensed the material contained in the five volumes of his elaborate treatise on Physiology. In this shape the large amount of information contained in the larger treatise is placed in a condition more readily to be reached and applied by the student or practitioner. The present volume is greatly enhanced in value by the introduction of over three hundred finely-executed illustrations of the text. Some of these cuts are original, but they are largely drawn from the works of Hirschfield Bernard, Sappey and Kolliker, together with others. Some few illustrations are also introduced taken from the admirable photographs made at the United States Army Medical Museum.

The volume of the work is not taken up with discussions of non-essential and undetermined points, but in a clear, straightforward manner endeavors to present to the reader what is known concerning the various points of human physiology.

The printing and general style of the work are excellent, and reflects credit upon the publishing house from which it is issued.

